# Factors associated with mobility decrease leading to disability: a cross-sectional nationwide study in Japan, with results from 8681 adults aged 20-89 years

**DOI:** 10.1186/s12877-021-02600-4

**Published:** 2021-11-19

**Authors:** Keiko Yamada, Satoshi Yamaguchi, Yoichi M. Ito, Takashi Ohe

**Affiliations:** 1 Departments of Sensory & Motor System Medicine, Faculty of Medicine, The University of Tokyo, Tokyo, Japan; 2 Department of Planning, Information and Management, The University of Tokyo Hospital, 7-3-1, Hongo, Bunkyo-ku, Tokyo, Japan; 3 Collage of Liberal Arts and Sciences, Chiba University, 1-8-1 Inohana, Chuo-ku, Chiba-shi, Chiba, Japan; 4 Data Science Center, Promotion Unit, Institute of Health Science Innovation for Medical Care, Hokkaido University Hospital, Kita 14-Jyo Nishi 5-chome, Kita-ku, Sapporo, Japan; 5 Department of Orhtopaedic Surgery, NTT Medical Center, 5-19-22, Higashigotanda, Shinagawa-ku, Tokyo Japan

**Keywords:** Mobility decrease, Disability, Cross-sectional study, All generations, Locomotive syndrome

## Abstract

**Background:**

Mobility decrease leading to disability can gradually develop during early life, however, its related factors are not well clarified. Therefore, we investigate the related factors of mobility decrease at various levels, using nationwide data in Japan.

**Methods:**

In total, 8681 independent community dwellers aged 20-89 years were analysed (average age, 51.6 years; 58.5% women). Three stages of mobility decrease were based on the locomotive syndrome risk test: Stage 1, emerging; Stage 2, progressing; Stage 3, progressed to restrict social engagement. Age was analysed using a simple quadratic function model.

**Results:**

The prevalence of Stages 1-3 was 31.6% (*n* = 2746), 5.8% (*n* = 504), and 3.2% (*n* = 278), respectively. On the multivariable logistic regression, increased age in participants aged ≥40 years (stage 1: odds ratio[OR] 1.05-1.20, stage 2: OR 1.04-1.22, stage 3: OR 1.05-1.22), female (stage 1: OR 2.28, 95% confidence interval [CI] 1.99-2.61, stage 2: OR 2.40, 95% CI 1.77-3.25, stage 3: OR 1.80, 95% CI 1.19-2.72), overweight status (stage 1: OR 1.56, 95% CI 1.34-1.82, stage 2: OR 3.19, 95% CI 2.38-4.27, stage 3: OR 2.87, 95% CI 1.90-4.32), hypertension (stage 1: OR 1.20, 95% CI 1.01-1.41, stage 2: OR 1.99, 95% CI 1.49-2.64, stage 3: OR 2.10, 95% CI 1.44-3.05), and diabetes mellitus (stage 1: OR 1.62, 95% CI 1.17-2.24, stage 2: OR 1.57, 95% CI 0.93-2.66, stage 3: OR 2.10, 95% CI 1.13-3.90) were positively associated. The frequency of physical activity/sports, even a few per month, was inversely associated with all stages (stage 1: OR 0.59-0.72, stage 2: OR 0.50-0.67, stage 3: 0.36-0.53). A one-year increase in age had a stronger impact on mobility decrease in older adults than in younger ones. Increased age in participants aged < 40 years and smoking were associated with Stage 1, while intake of various foods was inversely associated with Stages 1 and 2.

**Conclusion:**

Increased age (< 40 years) was associated with emerging mobility decrease, while that (≥ 40 years) was associated with any levels of mobility decrease. Female, lifestyle habits, including physical activities and overweight status, were associated with mobility decrease at every level.

**Supplementary Information:**

The online version contains supplementary material available at 10.1186/s12877-021-02600-4.

## Background

Disability degrades the quality of life and increases mortality, especially among older adults [1, 2]. The number of disabled older adults and the expenditure of long-term care insurance have significantly surged in Japan compared with 20 years ago [3]. The prevalence of disability is estimated to be one-fifth in Japanese older adults (≥ 65 years )[3, 4]. Known as a key factor of disability [1, 5–7], mobility decrease is an important urgent issue that must be addressed in Japan due to its rapidly super-aging society. As life expectance has increased worldwide, mobility decrease has become a global challenge [8]. Therefore, it is crucial to devise feasible strategies for addressing it, since it may can start in early life [5, 9–12].

The Japanese Orthopedic Association (JOA) proposed the concept of locomotive syndrome, a condition of decreased mobility essential to everyday life (e.g., walking, climbing stairs, or standing up from a chair) [13, 14]. The JOA developed the locomotive syndrome risk test to quantify mobility decrease; this test is a simple and easily feasible screening tool specialized for mobility decrease leading to disability and can continuously quantify mobility decrease from young/middle-aged to older adults [14–17]. Herein, disability is assumed to be certified as requiring care under the long-term care insurance in Japan [18]. The locomotive syndrome risk test is composed of two physical tests and one self-administrated questionnaire [14–17]. All three tests are recommended to complete, since they represent different dimension of mobility. Based on the indices of the test set, mobility decrease can be divided into three stages: emerging (Stage 1), progressing (Stage 2), and progressed to restrict social engagement (Stage 3) [14, 19]. According to the JOA, different measures are necessary against each mobility decrease stage, such as appropriate exercise and intake of nutrition for Stage 1, and medical examination or treatment for Stage 3 [14, 19].

In order to implement effective measures against mobility decrease leading to disability based on the stage, it is imperative to determine the related factors of all mobility decrease stages. A variety of predictors for mobility decrease have been examined, including age, sex, comorbidity, lifestyle factors (i.e., sedentary lifestyle, smoking, obesity), and physiological factors (i.e., poor nutritional status) [20], since mobility decrease is a compound of biological mechanisms and behavioural factors [21]. Similarly, many factors associated with disability have been reported, such as age, sex, obesity, smoking, insufficient physical activity, cardiovascular diseases, diabetes mellitus, and musculoskeletal diseases [22–26]. Disability and mobility decrease share similar factors, however, the factors associated with ‘mobility decrease leading to disability’ are not well clarified. In addition, most studies investigating factors associated with mobility decrease or disability have focused on older adults, rather than a wide range of age groups, even though mobility decrease can gradually develop during early life [5, 10–12].

Hence, this study investigates the related factors common to every stage of mobility decrease in independent community dwellers aged 20-89 years, using the locomotive syndrome risk test set, and determined whether related factors of mobility decrease differ with stage progression compared to that in participants without symptoms of mobility decrease.

## Methods

### Participants

Participants aged 20-89 years were recruited nationwide from 2017 to 2019, in proportion to the population of seven areas in Japan. The details of the participant recruiting method have been previously reported [10]. Briefly, participants comprised independent community dwellers who could walk independently without a caregiver’s help, and respond to self-administrated questionnaires. Individuals with certified need for long-term care insurance in Japan were excluded, since they are certified as “disabled”. Other exclusion criteria were related to temporary mobility decrease (since the mobility status could be worse than usual and easily fluctuate): a medical history of hospitalization within a month, history of trauma or surgeries of the spine/lower extremities within three months, and those undergoing treatment of the spine/lower extremities due to pain. All participants provided written informed consent and this study was approved by the authors’ affiliated institutions.

We reviewed the data of 10,444 individuals, of which 1400 were excluded according to the exclusion criteria. Of the remaining 9044, we analysed the data of 8681 community dwellers aged 20–89 years (3607 men, 5074 women) who completed all three tests of the locomotive syndrome risk test. In addition to the locomotive syndrome risk test results, we evaluated age, sex, height, weight, occupation, physical activities, nutrition, smoking, comorbidities, and a history of orthopaedic surgery.

### Locomotive syndrome risk test

The locomotive syndrome risk test comprises three tests, summarized below as previously described [15–17, 27, 28]. The validity, reliability, and feasibility of this test have been confirmed in previous studies [17, 28].

#### Two-step test

The two-step test assesses horizontal mobility, such as walking. The two-step test score is a standardized value adjusted for height, defined by the following equation:

$$\text{two-step}\ \text{test}\ \text{score}=\text{maximum}\ \text{two-step}\ \text{stride}\ \text{length}\ \left(\text{cm}\right)\div \text{individual}'\text{s}\ \text{height}\ \left(\text{cm}\right).$$

The two-step test score is strongly correlated with the maximum walking speed [16].

#### Stand-up test

The stand-up test assesses vertical mobility, such as standing up. The stand-up test score is a record of the lowest height (10, 20, 30, or 40 cm) of the stool from which the subject can successfully stand up (the subject is able to stand up using the stool on one or both legs and maintain their posture for 3 s). Supplementary Table 1 lists the scoring system of the stand-up test; higher scores indicate better ability. The stand-up test score is strongly associated with knee extensor strength [15, 29].

#### 25-question geriatric locomotive function scale [GLFS-25]

The GLFS-25 is a self-administrated questionnaire, indicating motor dysfunction [17]. The questionnaire comprises 25 items, each with a score of 0-4, on body pain, usual care, social activities, movement difficulties, and mental health status. Full and zero scores indicate the worst and best subjective locomotive conditions, respectively.

#### Stages of locomotive syndrome

Three stages of mobility decrease were defined based on the three locomotive syndrome risk test results (Supplementary Table 2) [14, 19]. Each test score had the threshold values for stages 1-3; the two-step test scores of 1.3, 1.1, 0.9, the stand-up test scores of 5, 3, 2 and the GLFS-25 scores of 7, 16, 24 correlated with stages 1, 2, 3, respectively. Participants were categorized into the highest (worst) stage determined by each test: for example, a participant with a two-step test score of 1.4, stand-up test score of 4, and GLFS-25 score of 20 was classified as Stage 2.

### Explanatory variables

Body mass index (BMI; kg/m^2^) was categorized into underweight (< 18.5), normal (18.5 ≤ BMI < 25), overweight (≥ 25) according to the conventional World Health Organization classification [30]. Subjects with obesity (BMI ≥ 30) were included in the overweight category, due to the low prevalence of obesity in the Japanese population (2-3%) [31]. Hypertension, diabetes mellitus, and dyslipidaemia were assessed by self-reported medication information. Occupation was classified into heavy, intermediate, light, other (miscellaneous activities), and unemployed, according to the National Health and Nutrition Examination (NHANES) study [32]. The frequency of physical activity/sports was categorized into four levels: rarely or never, a few times per month, a few times per week, and almost every day [33]. Nutrition was evaluated by dietary habits using food frequency scores, which covers the frequency per week of ten main food groups: meat, fish and shellfish, eggs, milk, soybean products, green and yellow vegetables, potatoes, fruits, seaweed, and fats and oils [34]. A score of 0-3 was assigned to each group, and the food frequency score was the sum of the scores of the 10 groups, ranging 0-30. Scores of 0 and 30 indicate the worst and best food intake frequency, respectively. Smoking was assessed as current smoker or not. Histories of other comorbidities (anaemia, heart diseases including angina pectoris and myocardial infarct, stroke, chronic kidney disease, and orthopaedic surgery) were judged as “yes” if the subject had been diagnosed as such by medical doctors.

### Statistical analysis

The prevalence rate and demographics of the participants were examined according to stage. All continuous variables are reported as the mean and standard deviation, whereas categorical variables are expressed as numbers and percentages. Differences in proportions among stages were evaluated using the chi-square test. Differences in continuous scores were evaluated using the analysis of variance. Using subjects without locomotive syndrome (Stage 0) as the reference, multivariable logistic regression analyses were performed to examine the associations between each stage and related factors. Exploratory variables comprised all those shown in Table 1, except for chronic kidney disease, which was excluded since its prevalence was < 1% (0.3%) in this study. Considering the possible quadratic effect of age on mobility decrease according to a previous study [9, 10], as well as the result of a restricted quadratic spline model [35] (Supplementary Fig. 1), a simple quadratic function model was adopted for age, since the effect of almost all the ages was sufficiently adjusted for by this model except for ages ≥80 years. The estimated odds ratio (OR) of a one-year change from the ages of 20, 30, 40, 50, 60, 70, and 80 years are presented as the representative values, since the effect of age differs due to its quadratic effect. Additionally, the effect of age was shown by plotting a fitted model on the log odds scale. A *p*-value < 0.05 was considered statistically significant. All statistical analyses were performed using SAS 9.4 (SAS Institute., NC, US).

**Table 1:** Background characteristics of the participants (*n* = 8681)

	none		Stage1		Stage2		Stage3		Total		P
N(%)	5153	(59.4)	2746	(31.6)	504	(5.8)	278	(3.2)	8681		
Age (years), mean ± SD	44.5	(16.1)	60.1	(15.7)	67.8	(15.1)	70.5	(14.7)	51.6	(18.2)	< 0.001
*20-64 (n, %)*	4369	(84.8)	1437	(52.3)	158	(31.4)	82	(29.5)	6046	(69.7)	
*65-74 (n, %)*	620	(12.0)	769	(28.0)	126	(25.0)	53	(19.1)	1568	(18.1)	
*75-89 (n, %)*	164	(3.2)	540	(19.7)	220	(43.7)	143	(51.4)	1067	(12.3)	
Sex											< 0.001
*Male*	2407	(46.7)	922	(33.6)	176	(34.9)	102	(36.7)	3607	(41.6)	
*Female*	2746	(53.3)	1824	(66.4)	328	(65.1)	176	(63.3)	5074	(58.5)	
BMI (Kg/m^2^)											< 0.001
*Underweight*	537	(10.4)	203	(7.4)	36	(7.1)	26	(9.4)	802	(9.2)	
*Normal*	3894	(75.5)	1970	(71.7)	311	(61.7)	183	(65.8)	6358	(73.2)	
*Overweight, Obese*	722	(14.0)	573	(20.9)	157	(31.2)	69	(24.8)	1521	(17.5)	
Hypertension (n, %)	478	(9.4)	692	(25.5)	220	(44.6)	131	(48.5)	1521	(17.8)	< 0.001
Diabetes mellitus (n, %)	88	(1.7)	137	(5.1)	42	(8.5)	31	(11.5)	298	(3.5)	< 0.001
Dyslipidaemia (n, %)	368	(7.2)	510	(18.8)	133	(27.0)	75	(27.8)	1086	(12.7)	< 0.001
Occupation (n, %)											< 0.001
*Heavy*	250	(4.9)	186	(6.8)	37	(7.4)	17	(6.2)	490	(5.7)	
*Intermediate*	1309	(25.8)	970	(35.7)	189	(37.9)	94	(34.4)	2562	(29.9)	
*Light*	2021	(39.8)	710	(26.1)	70	(14.0)	32	(11.7)	2833	(33.0)	
*Unemployed*	312	(6.1)	479	(17.6)	146	(29.3)	103	(37.7)	1040	(12.1)	
*Miscellaneous*	1189	(23.4)	376	(13.8)	57	(11.4)	27	(9.9)	1649	(19.2)	
Sports club membership (n, %)	1014	(19.8)	552	(20.2)	108	(21.4)	46	(16.6)	1720	(19.9)	0.41
Frequency of physical activity/sports (n, %)											< 0.001
*Rarely or Never*	1989	(38.7)	1113	(40.7)	203	(40.4)	125	(45.5)	3430	(39.6)	
*A few times/ month*	1029	(20.0)	382	(14.0)	57	(11.3)	27	(9.8)	1495	(17.3)	
*A few times/ week*	1377	(26.8)	670	(24.5)	128	(25.5)	73	(26.6)	2248	(26.0)	
*Almost Everyday*	751	(14.6)	571	(20.9)	115	(22.9)	50	(18.2)	1487	(17.2)	
Food frequency score, mean ± SD	18.4	(5.0)	19.2	(5.0)	19.4	(5.0)	19.7	(5.2)	18.7	(5.0)	< 0.001
Smoking (n, %)	597	(11.6)	275	(10.1)	34	(6.8)	19	(6.9)	925	(10.7)	< 0.001
Anaemia (n, %)	679	(13.2)	411	(15.0)	75	(14.9)	53	(19.1)	1218	(14.1)	0.010
History of heart diseases (n, %)	79	(1.5)	135	(4.9)	46	(9.1)	30	(10.8)	290	(3.4)	< 0.001
History of stroke (n, %)	27	(0.5)	55	(2.0)	19	(3.8)	22	(7.9)	123	(1.4)	< 0.001
Chronic kidney disease (n, %)	5	(0.1)	11	(0.4)	4	(0.8)	2	(0.8)	22	(0.3)	< 0.001
History of orthopaedic surgery (n, %)	510	(10.8)	384	(15.5)	93	(20.6)	76	(30.2)	1063	(13.5)	< 0.001

*SD* standard deviation, *BMI* body mass index

## Results

Summary characteristics of the participants according to stage are shown in Table 1. Of the 8681 participants, the prevalence of Stages 1-3 was 31.6% (*n* = 2746), 5.8% (*n* = 504), and 3.2% (*n* = 278), respectively. Stage 3 had the highest mean age (*p* <  0.001) and the highest rates of rare or no physical activity/sports (p <  0.001) and comorbidities such as hypertension and diabetes mellitus (p <  0.001), and the highest food frequency scores (p <  0.001).

**Fig. 1:**
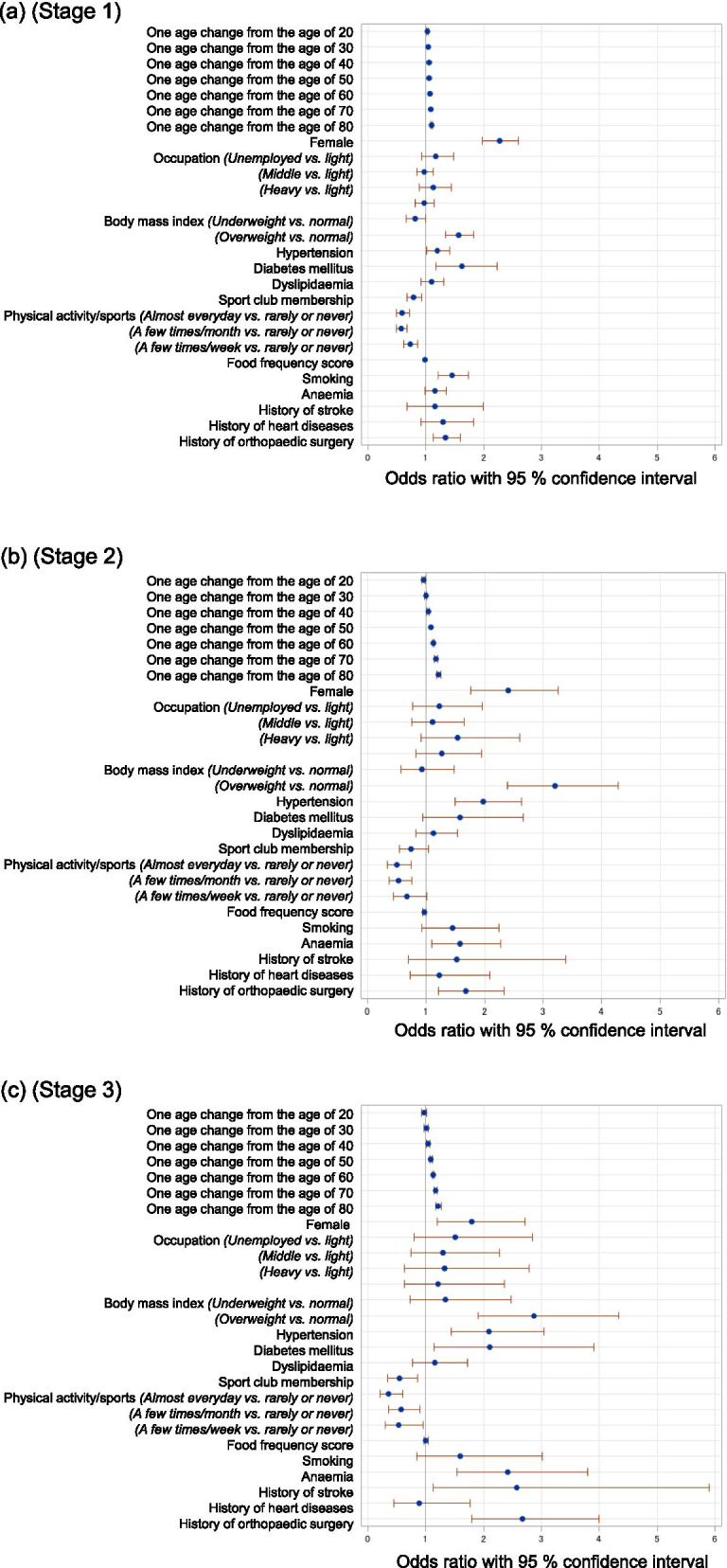
The odds ratios (ORs) for Stages 1-3 mobility decrease (a-c, respectively). The odds ratios for each stage of mobility decrease with Stage 0 as the reference were shown in the Fig. 1. Circles indicate point estimates compared to the reference. The width of the horizontal lines represents the 95% confidence intervals (CIs) for each explanatory variable

**Fig. 2:**
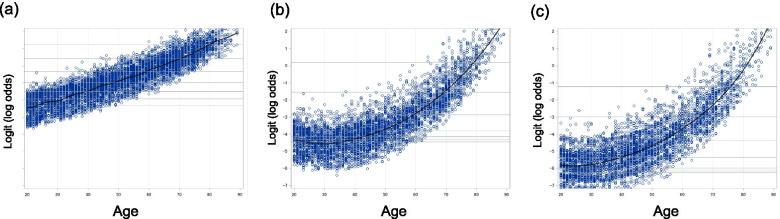
The odds ratios (ORs) for one-year increments in age for stage 1-3 (**a**-**c**, respectively). Plots of the fitted model on the log odds scale, with reference lines of the estimate of the log odds, at the ages of 20, 30, 40, 50, 60, 70, and 80 years are shown for each stage. In Stage 1 (**a**), the fitted curve shows a comparatively linear increase, while in Stages 2 (**b**) and 3 (**c**), the fitted curve starts to sharply increase above the age of 40 years

The results of the multivariable logistic regression analysis for Stages 1-3, with age and age-squared terms in the exploratory variables, are shown in Table 2 and Fig. 1a-c. Increased age in participants aged ≥40 years, female sex, overweight status, hypertension, frequency of physical activity/sports, and a history of orthopaedic surgery were related factors in all stages. Among these, the frequency of physical activity/sports was inversely associated with all stages. Although sports club membership and diabetes mellitus also showed association with all stages, the relationship failed to reach significance for Stage 2. In contrast, dyslipidaemia was not associated with any stages. Furthermore, increased age in participants aged < 40 years was associated with Stage 1, but not Stages 2 and 3. The trajectories of the increase in estimated OR with a one-year change in age differed between Stage 1 and Stages 2 and 3 (Fig. 2 a-c). Smoking was associated with only Stage 1 (OR 1.45, 95% confidence interval [CI] 1.20-1.74), while nutrition (frequency food score) was inversely associated with Stage 1 (OR 0.98, 95% CI 0.97-0.99) and Stage 2 (OR 0.97, 95% CI 0.94-0.99), but not Stage 3. The higher the stage, the greater the odds ratio for anaemia, which reached statistical significance in Stages 2 (OR 1.58, 95% CI 1.10-2.27) and 3 (OR 2.41, 95% CI 1.54-3.78).

**Table 2:** Multivariable logistic regression analysis for stages 1-3 of the locomotive syndrome

	Stage1			Stage2			Stage3		
	OR	95%CI	P	OR	95%CI	P	OR	95%CI	P
Age (years old)									
*one year change from the age of 20*	1.03	1.02-1.05	< 0.001	0.96	0.92-0.99	< 0.001	0.97	0.92-1.02	0.23
*one year change from the age of 30*	1.04	1.03-1.05	< 0.001	0.99	0.97-1.02	0.64	1.01	0.97-1.05	0.72
*one year change from the age of 40*	1.05	1.04-1.06	< 0.001	1.04	1.02-1.05	< 0.001	1.05	1.02-1.07	< 0.001
*one year change from the age of 50*	1.06	1.06-1.07	< 0.001	1.08	1.07-1.09	< 0.001	1.09	1.07-1.11	< 0.001
*one year change from the age of 60*	1.07	1.07-1.08	< 0.001	1.12	1.11-1.14	< 0.001	1.13	1.11-1.15	< 0.001
*one year change from the age of 70*	1.08	1.07-1.10	< 0.001	1.17	1.14-1.19	< 0.001	1.17	1.14-1.20	< 0.001
*one year change from the age of 80*	1.20	1.16-1.23	< 0.001	1.22	1.18-1.25	< 0.001	1.22	1.17-1.26	< 0.001
Sex									
*Male*	Ref			Ref			Ref		
*Female*	2.28	1.99-2.61	< 0.001	2.40	1.77-3.25	< 0.001	1.80	1.19-2.72	0.005
Occupation									
*Heavy*	1.13	0.88-1.44	0.34	1.54	0.91-2.60	0.11	1.32	0.63-2.78	0.47
*Middle*	0.98	0.84-1.13	0.74	1.12	0.76-1.66	0.57	1.30	0.74-2.28	0.36
*Light*	Ref			Ref			Ref		
*Unemployed*	1.17	0.92-1.48	0.19	1.24	0.78-1.98	0.37	1.51	0.80-2.87	0.20
*Miscellaneous*	0.96	0.81-1.15	0.68	1.27	0.83-1.96	0.27	1.21	0.62-2.36	0.57
BMI (Kg/m^2^)									
*Underweight*	0.81	0.65-1.00	0.045	0.92	0.57-1.47	0.72	1.34	0.73-2.46	0.35
*Normal*	Ref			Ref			Ref		
*Overweight, Obese*	1.56	1.34-1.82	< 0.001	3.19	2.38-4.27	< 0.001	2.87	1.90-4.32	< 0.001
Hypertension	1.20	1.01-1.41	0.035	1.99	1.49-2.64	< 0.001	2.10	1.44-3.05	< 0.001
Diabetes mellitus	1.62	1.17-2.24	0.004	1.57	0.93-2.66	0.090	2.10	1.13-3.90	0.019
Dyslipidaemia	1.09	0.91-1.30	0.34	1.12	0.82-1.53	0.48	1.15	0.76-1.72	0.51
Sports club membership	0.79	0.67-0.92	0.003	0.75	0.54-1.04	0.080	0.55	0.35-0.86	0.009
Frequency of physical activity/sports									
*Rarely or Never*	Ref			Ref			Ref		
*A few times/ month*	0.72	0.61-0.86	< 0.001	0.67	0.45-1.00	0.052	0.53	0.29-0.95	0.032
*A few times/ week*	0.57	0.48-0.67	< 0.001	0.53	0.37-0.75	< 0.001	0.57	0.36-0.89	0.014
*Almost Everyday*	0.59	0.49-0.71	< 0.001	0.50	0.35-0.74	< 0.001	0.36	0.21-0.60	< 0.001
Food frequency score	0.98	0.97-0.99	0.004	0.97	0.94-0.99	0.015	1.00	0.97-1.04	0.81
Smoking	1.45	1.20-1.74	< 0.001	1.44	0.93-2.25	0.11	1.59	0.84-3.00	0.15
Anaemia	1.15	0.98-1.36	0.087	1.58	1.10-2.27	0.014	2.41	1.54-3.78	< 0.001
History of stroke	1.15	0.67-1.98	0.62	1.52	0.69-3.35	0.30	2.56	1.12-5.87	0.027
History of heart diseases	1.32	0.94-1.86	0.11	1.27	0.75-2.18	0.38	0.91	0.46-1.82	0.79
History of orthopaedic surgery	1.34	1.13-1.59	0.001	1.68	1.21-2.33	0.002	2.67	1.79-3.99	< 0.001

*OR* Odds ratio, *CI* confidence interval, *Ref* reference, *BMI* body mass index

## Discussion

In the present study, we investigated the factors associated with mobility decrease leading to disability among independent community dwellers aged 20-89 years, using nationwide data in Japan. Increased age in participants aged ≥40, female, overweight status, hypertension, and diabetes mellitus were positively associated with Stages 1-3 mobility decrease, while sports club membership and the frequency of physical activities/sports were inversely associated with Stages 1-3. Increased age in participants aged < 40 years and smoking were associated with Stage 1. Nutrition (food frequency score) was inversely associated with Stages 1 and 2, while anaemia was associated with Stages 2 and 3.

Our findings showed that increased age, especially > 40 years, was a related factor for any level of mobility decrease, in line with previous studies reporting age-dependent physical ability as related to mobility measures, such as grip power, muscle power of the lower extremities, or usual and maximum gait speed [5, 9–12]. Moreover, our study also showed that a one-year increase in age had a stronger impact on mobility decrease in older participants than in younger participants. In addition, the results showed that increased age was related with Stage 1 mobility decrease, even in participants aged < 40 years, which supports the concept that mobility decrease gradually starts early in life. Thus, education regarding mobility decrease in younger generations is necessary for the prevention of future mobility decrease.

The present study found that females were at greater risk of mobility decrease than males, which is consistent with previous studies indicating a life-long female disadvantage on physical performance tests, with no specific risk groups [36, 37], as well as in mobility difficulties [25]. Considering the prevalence of disability is higher in females than in males [4, 38], strategies to prevent mobility decrease targeting women are desired.

Overweight status, diabetes mellitus, and hypertension were associated with almost every level of mobility decrease. Previous studies have reported that increased components of Mets (obesity, hypertension, elevated fasting glucose, and dyslipidaemia) are likely to be associated with mobility limitations among older adults [39, 40]. The present study additionally showed that diabetes, overweight status, and hypertension are associated with every level of mobility decrease; however, dyslipidaemia was not associated with any level of mobility decrease. Several studies reported the association between dyslipidaemia and mobility decrease, whereas a study from US reported its association might be sex-dependent (only in females) [39–41]. Further research is warranted to clarify the association between dyslipidaemia and mobility decrease.

Interestingly, underweight status was positively associated with stage 3, while it was negatively associated with stage 1. These different results may be attributed to the age distribution of the stages 1 and 3. Most of stage 3 comprised older adults, in which underweight was reported to be risk to functional decline and sarcopenia [42, 43]. Meanwhile, half of the stage 1 group were young/middle-aged adults. The association between underweight and emerging mobility decrease in these population has not been well clarified, however, younger adults who are underweight might easily stand up or walk with less energetic cost.

The level of physical activity and sports club membership were inversely related to every level of mobility decrease, in accordance with a previous study reporting that regular physical activity modified the age-related decline in mobility among community dwellers [44]. Furthermore, the present study revealed that individuals with even a few physical activities per month had a lower risk of mobility decrease than those with rare or no physical activity despite the recommendations of the World Health Organization (i.e., as much physical activity as possible, and moderate-intensity physical activity of ≥150 min per week or ≥ 75 min of vigorous-intensity activities a week for good health) [45]. Since physical activity is a modifiable lifestyle habit, the finding that any frequency of physical activity is inversely related with mobility decrease, compared to that with no or rare activity, could be an encouraging take-home message to the public. However, further investigations are necessary to confirm the effect of the frequency of physical activity on mobility decrease leading to disability.

Increased dietary variety was inversely related to Stages 1 and 2, although the correlations were relatively small. Higher dietary variety is reportedly associated with better mobility and is a predictive factor of a smaller decline in mobility; additionally, some dietary patterns may play a preventive role against mobility disability among older adults [46, 47]. The present study showed that the intake of various food groups may modify Stages 1 and 2 mobility decrease, which might result in substantial differences in long-term mobility.

Smoking was associated with Stage 1 mobility decrease, which is consistent with reports that smokers may have structural and metabolic damage in their muscles and lower physical functioning compared to that in non-smokers [48, 49]. However, the present study indicated that smoking was not associated with Stages 2 and 3, which may result from the complexity of mobility decrease. The more mobility decrease is progressed, the more other covariates not included in this study might have effects on mobility decrease. Further investigations are warranted to confirm whether there is an effect of smoking on progressed mobility decrease.

The present study found that anaemia was positively associated with mobility decrease. Moreover, as the level of mobility deteriorated, anaemia had stronger impact on the dependent variable. We could not determine whether anaemia is a result or a cause of mobility decrease, since anaemia can impair physical performance via restricting oxygen supply to skeletal muscles, while immobility causes anaemia by adipocyte accumulation in bone marrow [50, 51]. Therefore, further investigation are warranted to clarify the causal relationship between anaemia and mobility decrease.

This study has several limitations. First, we could not determine the causal effects between the related factors and mobility decrease, since this was a cross-sectional study. Further detailed studies or analyses are necessary to draw conclusions regarding the causal relationship between related factors examined in this study and mobility decrease. Second, other possible factors may influence mobility decrease, such as mental status (including a history of depression or cognitive impairment), visual defects, hearing loss, trauma without orthopaedic surgery or socioeconomic status. Third, history of comorbidities were self-reported. Forth, the participants of this study could have potential biases, as some were recruited from public medical check-ups, and others were participants of another cohort study. Generalizations to the general population should be made cautiously, since the participants of this study were “individuals who could walk by themselves without a caregiver’s support”, which means that they could be healthier than the general population, especially in older adults. While performing locomotive syndrome risk tests for older adults with disability, we must consider safety implementation.

## Conclusions

Increased age (< 40 years) was related with emerging mobility decrease, while that (≥ 40 years) was associated with any levels of mobility decrease. Education regarding mobility decrease for all generations as well as interventions targeting females are essential for healthy aging. The effect of lifestyle habits such as physical activities or overweight status should be further investigated.

## Supplementary Information

**Additional file 1: Supplementary Figure 1**. The effect of age using the restricted quadratic spline and the simple quadratic function model. Smoothing component for Stages 1-3, with 95% confidence intervals of the restricted quadratic spline and adjusted logits of the simple quadratic function model are shown in these figures. The two columns on the left show the results of the smoothing component panel for each stage. We performed this analysis using restricted quadratic spline to verify whether age had a quadratic effect or not. The far-right column shows the relationship between age and adjusted logit by exploratory variables other than age and age squared term. These results show that the effect of almost all the ages was sufficiently adjusted for by the simple quadratic function model, except for ages ≥80 years. GCV, generalized cross validation; df, degree of freedom.

**Additional file 2: Supplementary Table 1**. Scoring system of the stand-up test.

**Additional file 3: Supplementary Table 2**. Stage of mobility decrease based on the scores of the three tests.

## Data Availability

The datasets used and/or analyzed during the current study are available from the corresponding author on reasonable request.

## Abbreviations

GLFS: Geriatric locomotive function scale
LTCI: Long-term care insurance
JOA: Japanese Orthopaedic Association

## References

[1] Wu LW, Chen WL, Peng TC, Chiang ST, Yang HF, Sun YS, Chan JY, Kao TW (2016). All-cause mortality risk in elderly individuals with disabilities: a retrospective observational study. BMJ Open.

[2] Collaborators GDaIIaP (2016). Global, regional, and national incidence, prevalence, and years lived with disability for 310 diseases and injuries, 1990-2015: a systematic analysis for the global burden of disease study 2015. Lancet.

[3] Long-term Care Insurance System of Japan. November 2016, Ministry of Health, Labour and Welfare, Japan. https://www.mhlw.go.jp/english/policy/care-welfare/care-welfare-elderly/dl/ltcisj_e.pdf Accessed: 21 May, 2021.

[4] Yoshida D, Ninomiya T, Doi Y, Hata J, Fukuhara M, Ikeda F, Mukai N, Kiyohara Y (2012). Prevalence and causes of functional disability in an elderly general population of Japanese: the Hisayama study. J Epidemiol.

[5] Ferrucci L, Cooper R, Shardell M, Simonsick EM, Schrack JA, Kuh D (2016). Age-related change in mobility: perspectives from life course epidemiology and Geroscience. J Gerontol A Biol Sci Med Sci.

[6] Akune T, Muraki S, Oka H, Tanaka S, Kawaguchi H, Tokimura F, Yoshida H, Suzuki T, Nakamura K, Yoshimura N (2014). Incidence of certified need of care in the long-term care insurance system and its risk factors in the elderly of Japanese population-based cohorts: the ROAD study. Geriatr Gerontol Int.

[7] Groessl EJ, Kaplan RM, Rejeski WJ, Katula JA, King AC, Frierson G, Glynn NW, Hsu FC, Walkup M, Pahor M (2007). Health-related quality of life in older adults at risk for disability. Am J Prev Med.

[8] Wan He DG, Kowal P (2016). An Aging World: 2015.

[9] Suetta C, Haddock B, Alcazar J, Noerst T, Hansen OM, Ludvig H, et al. The Copenhagen sarcopenia study: lean mass, strength, power, and physical function in a Danish cohort aged 20-93 years. J Cachexia Sarcopenia Muscle. 2019;10(6):1316–29. 10.1002/jcsm.12477 PMC6903448 31419087

[10] Yamada K, Ito YM, Akagi M, Chosa E, Fuji T, Hirano K, Ikeda S, Ishibashi H, Ishibashi Y, Ishijima M (2020). Reference values for the locomotive syndrome risk test quantifying mobility of 8681 adults aged 20-89 years: a cross-sectional nationwide study in Japan. J Orthop Sci.

[11] Bohannon RW (1997). Comfortable and maximum walking speed of adults aged 20-79 years: reference values and determinants. Age Ageing.

[12] Samson MM, Meeuwsen IB, Crowe A, Dessens JA, Duursma SA, Verhaar HJ (2000). Relationships between physical performance measures, age, height and body weight in healthy adults. Age Ageing.

[13] Nakamura K, Ogata T (2016). Locomotive syndrome: definition and management. Clin Rev Bone Miner Metab.

[14] Locomotive Challenge Council. Locomotive syndrome. In: Locomotive Challenge Council, ed. Locomotive syndrome pamphlet 2015. Tokyo: Japanese Orthopaedic Association; 2015. https://locomo-joa.jp/assets/pdf/index_english.pdf. Accessed: 1 June, 2021.

[15] Muranaga S (2001). Evaluation of the muscular strength of the lower extremities using the standing movement and clinical application. J Showa Med Assoc.

[16] Muranaga S, Hirano K (2003). Development of a convenient way to predict ability to walk, using a two-step test. J Showa Med Assoc.

[17] Seichi A, Hoshino Y, Doi T, Akai M, Tobimatsu Y, Iwaya T (2012). Development of a screening tool for risk of locomotive syndrome in the elderly: the 25-question geriatric locomotive function scale. J Orthop Sci.

[18] Yamada K, Muranaga S, Shinozaki T, Nakamura K, Tanaka S, Ogata T (2018). Age independency of mobility decrease assessed using the locomotive syndrome risk test in elderly with disability: a cross-sectional study. BMC Geriatr.

[19] Locomotive Challenge Council. Locomotive syndrome. In: Locomotive Challenge Council, ed. Locomotive syndrome pamphlet 2020. Tokyo: Japanese Orthopaedic Association; 2020 (In Japanese).

[20] Yeom HA, Fleury J, Keller C (2008). Risk factors for mobility limitation in community-dwelling older adults: a social ecological perspective. Geriatr Nurs.

[21] Anton SD, Cruz-Almeida Y, Singh A, Alpert J, Bensadon B, Cabrera M, Clark DJ, Ebner NC, Esser KA, Fillingim RB (2020). Innovations in Geroscience to enhance mobility in older adults. Exp Gerontol.

[22] Taş U, Verhagen AP, Bierma-Zeinstra SM, Hofman A, Odding E, Pols HA, Koes BW (2007). Incidence and risk factors of disability in the elderly: the Rotterdam study. Prev Med.

[23] Manton KG (2008). Recent declines in chronic disability in the elderly U.S. population: risk factors and future dynamics. Annu Rev Public Health.

[24] Fancourt D, Steptoe A (2019). Comparison of physical and social risk-reducing factors for the development of disability in older adults: a population-based cohort study. J Epidemiol Community Health.

[25] Wray LA, Blaum CS (2001). Explaining the role of sex on disability: a population-based study. Gerontologist.

[26] Amiri S, Behnezhad S (2020). Smoking and disability pension: a systematic review and meta-analysis. Public Health.

[27] Yoshimura N, Muraki S, Oka H, Tanaka S, Ogata T, Kawaguchi H, Akune T, Nakamura K (2015). Association between new indices in the locomotive syndrome risk test and decline in mobility: third survey of the ROAD study. J Orthop Sci.

[28] Ogata T, Muranaga S, Ishibashi H, Ohe T, Izumida R, Yoshimura N, Iwaya T, Nakamura K (2015). Development of a screening program to assess motor function in the adult population: a cross-sectional observational study. J Orthop Sci.

[29] Miyatake N, Fujii M, Nishikawa H, Wada J, Shikata K, Makino H, Kimura I (2000). Clinical evaluation of muscle strength in 20-79-years-old obese Japanese. Diabetes Res Clin Pract.

[30] Executive summary of the clinical guidelines on the identification, evaluation, and treatment of overweight and obesity in adults. Arch Intern Med. 1998;158(17):1855–67. 10.1001/archinte.158.17.1855 9759681

[31] Tsugane S (2012). Alcohol, smoking, and obesity epidemiology in Japan. J Gastroenterol Hepatol.

[32] Steeves JA, Tudor-Locke C, Murphy RA, King GA, Fitzhugh EC, Harris TB (2015). Classification of occupational activity categories using accelerometry: NHANES 2003-2004. Int J Behav Nutr Phys Act.

[33] Ministry of Education Culture, Sports, Science and Technology (MEXT), Japan: Report on Physical and Motor Ability survey (In Japanese). https://www.mext.go.jp/a_menu/sports/stamina/03040901.htm. Accessed 08 Nov 2021.

[34] Kimura M, Moriyasu A, Kumagai S, Furuna T, Akita S, Kimura S, Suzuki T (2013). Community-based intervention to improve dietary habits and promote physical activity among older adults: a cluster randomized trial. BMC Geriatr.

[35] Howe CJ, Cole SR, Westreich DJ, Greenland S, Napravnik S, Eron JJ (2011). Splines for trend analysis and continuous confounder control. Epidemiology.

[36] Sialino LD, Schaap LA, van Oostrom SH, Nooyens ACJ, Picavet HSJ, Twisk JWR, Verschuren WMM, Visser M, Wijnhoven HAH (2019). Sex differences in physical performance by age, educational level, ethnic groups and birth cohort: the longitudinal aging study Amsterdam. PLoS One.

[37] Wheaton FV, Crimmins EM (2016). Female disability disadvantage: a global perspective on sex differences in physical function and disability. Ageing Soc.

[38] Statistics on Long-term Care Insurance Recipients (2019), Ministry of Health, Labour and Welfare, Japan. https://www.mhlw.go.jp/toukei/saikin/hw/kaigo/kyufu/19/dl/02.pdf Accessed: 5 Oct, 2021. (In Japanese).

[39] Liaw FY, Kao TW, Wu LW, Wang CC, Yang HF, Peng TC, Sun YS, Chang YW, Chen WL (2016). Components of metabolic syndrome and the risk of disability among the elderly population. Sci Rep.

[40] Penninx BW, Nicklas BJ, Newman AB, Harris TB, Goodpaster BH, Satterfield S, de Rekeneire N, Yaffe K, Pahor M, Kritchevsky SB (2009). Metabolic syndrome and physical decline in older persons: results from the health, aging and body composition study. J Gerontol A Biol Sci Med Sci.

[41] Okoro CA, Zhong Y, Ford ES, Balluz LS, Strine TW, Mokdad AH (2006). Association between the metabolic syndrome and its components and gait speed among U.S. adults aged 50 years and older: a cross-sectional analysis. BMC Public Health.

[42] Chen CM, Chang WC, Lan TY (2015). Identifying factors associated with changes in physical functioning in an older population. Geriatr Gerontol Int.

[43] Miura H, Sakaguchi K, Ogawa W, Tamori Y (2021). Clinical features of 65-year-old individuals in Japan diagnosed with possible sarcopenia based on the Asian working Group for Sarcopenia 2019 criteria. Geriatr Gerontol Int.

[44] Landi F, Calvani R, Picca A, Tosato M, Martone AM, D'Angelo E, Serafini E, Bernabei R, Marzetti E (2018). Impact of habitual physical activity and type of exercise on physical performance across ages in community-living people. PLoS One.

[45] Bull FC, Al-Ansari SS, Biddle S, Borodulin K, Buman MP, Cardon G, Carty C, Chaput JP, Chastin S, Chou R (2020). World Health Organization 2020 guidelines on physical activity and sedentary behaviour. Br J Sports Med.

[46] Yokoyama Y, Nishi M, Murayama H, Amano H, Taniguchi Y, Nofuji Y, Narita M, Matsuo E, Seino S, Kawano Y (2017). Dietary variety and decline in lean mass and physical performance in community-dwelling older Japanese: a 4-year follow-up study. J Nutr Health Aging.

[47] Agarwal P, Wang Y, Buchman AS, Bennett DA, Morris MC. Dietary patterns and self-reported incident disability in older adults. J Gerontol A Biol Sci Med Sci. 2019;74(8):1331–7. 10.1093/gerona/gly211 PMC6625581 30247552

[48] Montes de Oca M, Loeb E, Torres SH, De Sanctis J, Hernández N, Tálamo C (2008). Peripheral muscle alterations in non-COPD smokers. Chest.

[49] van den Borst B, Koster A, Yu B, Gosker HR, Meibohm B, Bauer DC, Kritchevsky SB, Liu Y, Newman AB, Harris TB (2011). Is age-related decline in lean mass and physical function accelerated by obstructive lung disease or smoking?. Thorax.

[50] Payne MW, Uhthoff HK, Trudel G (2007). Anemia of immobility: caused by adipocyte accumulation in bone marrow. Med Hypotheses.

[51] Penninx BW, Guralnik JM, Onder G, Ferrucci L, Wallace RB, Pahor M (2003). Anemia and decline in physical performance among older persons. Am J Med.

