# Supplementary material for: Factors associated with mobility decrease leading to disability: a cross-sectional nationwide study in Japan, with results from 8681 adults aged 20-89 years
Source: BMC Geriatr. 2021 Nov 19;21:651. doi: 10.1186/s12877-021-02600-4 (PMC8603520; doi:10.1186/s12877-021-02600-4)
Supplement: Supplementary file 1 — Additional file 1: Supplementary Figure 1. The effect of age using the restricted quadratic spline and the simple quadratic function model. Smoothing component for Stages 1-3, with 95% confidence intervals of the restricted quadratic spline and adjusted logits of the simple quadratic function model are shown in these figures. The two columns on the left show the results of the smoothing component panel for each stage. We performed this analysis using restricted quadratic spline to verify whether age had a quadratic effect or not. The far-right column shows the relationship between age and adjusted logit by exploratory variables other than age and age squared term. These results show that the effect of almost all the ages was sufficiently adjusted for by the simple quadratic function model, except for ages ≥80 years. GCV, generalized cross validation; df, degree of freedom. [file 12877_2021_2600_MOESM1_ESM.pdf]

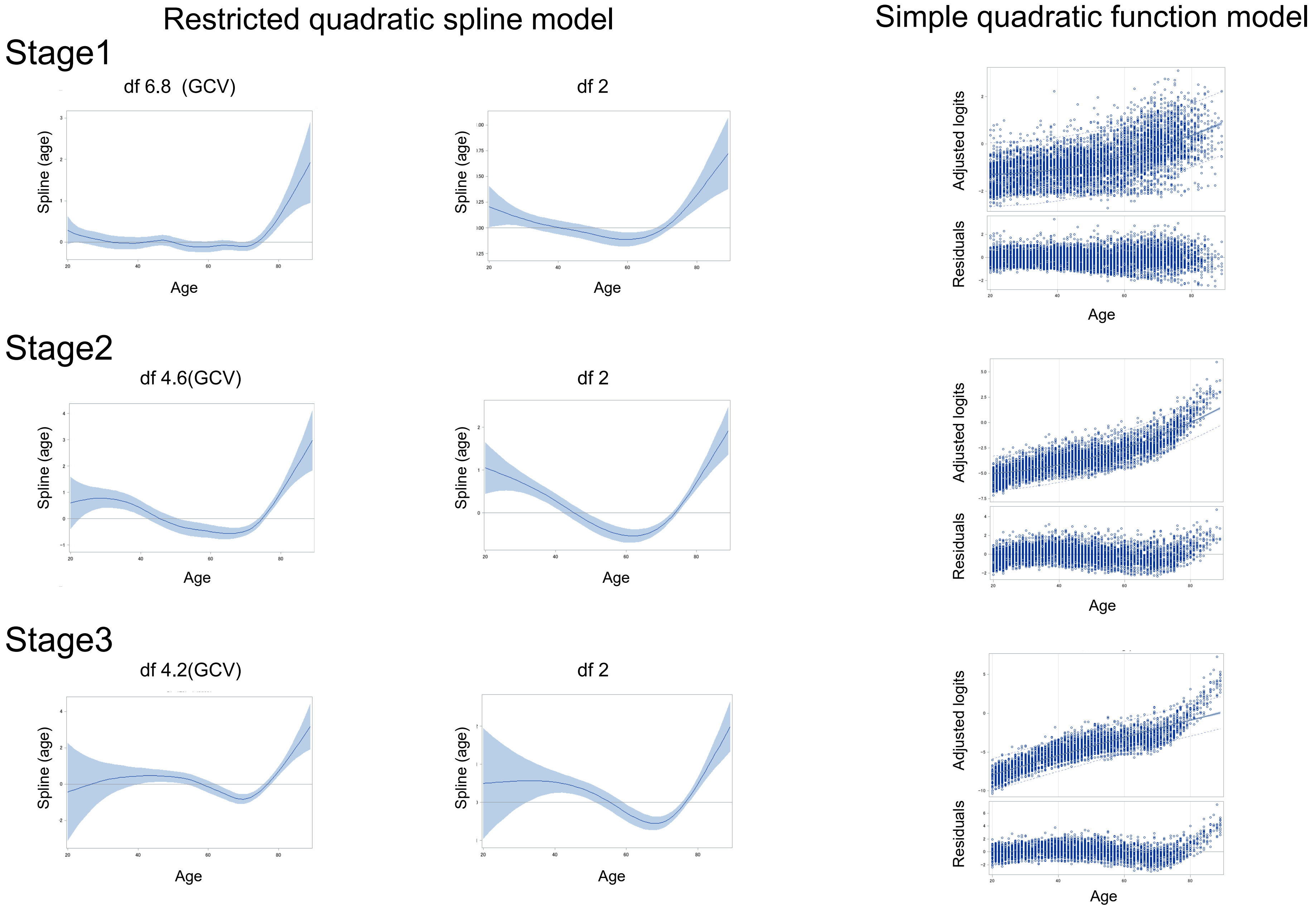
Restricted quadratic spline model
Simple quadratic function model
Stage1
df 6.8 (GCV)
df 2
Stage2
df 4.6(GCV)
df 2
Stage3
df 4.2(GCV)
df 2
Spline (age)
Age
Adjusted logits
Residuals
