# Supplementary material for: Factors associated with mobility decrease leading to disability: a cross-sectional nationwide study in Japan, with results from 8681 adults aged 20-89 years
Source: BMC Geriatr. 2021 Nov 19;21:651. doi: 10.1186/s12877-021-02600-4 (PMC8603520; doi:10.1186/s12877-021-02600-4)
Supplement: Supplementary file 2 — Additional file 2: Supplementary Table 1. Scoring system of the stand-up test. [file 12877_2021_2600_MOESM2_ESM.docx]

Supplementary table 1. Scoring system of the stand-up test

|  | **Two-leg stand** | | | | | **One-leg stand** | | | |
| --- | --- | --- | --- | --- | --- | --- | --- | --- | --- |
| **Height** | **Fail at** | **40 cm** | **30 cm** | **20 cm** | **10 cm** | **40 cm** | **30 cm** | **20 cm** | **10 cm** |
|  | **40 cm** |  |  |  |  |  |  |  |  |
| Score | 0 | 1 | 2 | 3 | 4 | 5 | 6 | 7 | 8 |

The one-leg stand requires subjects to succeed at the indicated height with both the *right* and *left* leg.
