# Supplementary material for: Factors associated with mobility decrease leading to disability: a cross-sectional nationwide study in Japan, with results from 8681 adults aged 20-89 years
Source: BMC Geriatr. 2021 Nov 19;21:651. doi: 10.1186/s12877-021-02600-4 (PMC8603520; doi:10.1186/s12877-021-02600-4)
Supplement: Supplementary file 3 — Additional file 3: Supplementary Table 2. Stage of mobility decrease based on the scores of the three tests. [file 12877_2021_2600_MOESM3_ESM.docx]

Supplementary table 2. Stage of mobility decrease based on the scores of the three tests

|  | **Stage 0 (Normal)** | **Stage 1** | **Stage 2** | **Stage 3** |
| --- | --- | --- | --- | --- |
| Two-step test score (TS) | 1.3≤TS | 1.1≤TS<1.3 | 0.9≤TS<1.1 | TS<0.9 |
| Stand-up test score (SU) | ≥5 | 3 or 4 | 2 | 0 or 1 |
| GLFS-25 score (GLFS) | 0≤GLFS<7 | 7≤GLFS<16 | 16≤GLFS<24 | 24≤GLFS |

GLFS, question geriatric locomotive function scale.

Participants were categorized into the highest stage indicated by the results of the three tests.
